# Patient-centered gastrointestinal function assessment technologies: a paradigm shift from traditional approaches to non-invasive innovations

**DOI:** 10.3389/fphys.2026.1776210

**Published:** 2026-03-12

**Authors:** Qian Zhu, Qian Li, Yan Zan, Yuchen Lu, Liangjun Xia, Youbing Xia, Tiancheng Xu

**Affiliations:** 1 Key Laboratory of Acupuncture and Medicine Research of Ministry of Education, Nanjing University of Chinese Medicine, Nanjing, China; 2 Xuzhou Medical University, Xuzhou, China; 3 Department of Traditional Chinese Medicine, Qinghai University Medical College, Qinghai University, Xining, China; 4 State Key Laboratory of Reproductive Medicine and Offspring Health, Nanjing Medical University, Nanjing, China

**Keywords:** clinical translation, gastrointestinal function assessment, non-invasive technology, patient-friendly, wireless motility capsule

## Abstract

Gastrointestinal function assessment has assumed an increasingly pivotal role in diagnosing and managing digestive system disorders, yet the widespread clinical application of conventional techniques remains constrained by ionizing radiation exposure, high procedural invasiveness, and heavy reliance on specialized equipment—limitations severely compromising patient acceptance and service accessibility. Guided by the patient-centered care paradigm, this paper systematically reviews the evolution of such assessment technologies, delineating the intrinsic limitations of radionuclide imaging, invasive manometry, and traditional electrogastrography in terms of safety, comfort, and accessibility, while synthesizing research progress and clinical evidence of non-invasive modalities like stable isotope breath tests, surface electrogastrographic mapping, wireless motility capsules, and wearable monitors. It also explores AI’s potential in integrating multi-dimensional physiological signals to support clinical decision-making and examines translational barriers from technical validation to routine practice. Accumulating evidence shows emerging non-invasive technologies provide clinically actionable insights while alleviating patient burden, but most current studies focus on diagnostic accuracy validation, lacking high-quality evidence for their benefits in guiding treatment or improving long-term prognosis. Future research should prioritize patient outcomes, rigorously assess technologies’ applicability and value in real-world settings, and facilitate innovation transformation from parameter measurement to patient-centric care and from laboratory to clinical practice.

## 1 Introduction

For a long time, the diagnosis of digestive system diseases has mainly focused on the identification of structural lesions. The continuous advancement of endoscopic and imaging techniques has significantly improved the detection ability of organic diseases such as tumors and ulcers. However, in clinical practice, a large number of patients with obvious digestive tract symptoms show no abnormalities in structural examinations (Harold et al., 2022; Cassinotti et al., 2023). The global prevalence of functional gastrointestinal disorders (FGIDs) is as high as 20%–40%, posing a public health challenge that cannot be ignored (Sperber et al., 2021). Traditional diagnostic approaches often rely on the “exclusion method”. Patients usually have to undergo a series of invasive examinations to rule out organic diseases one by one before receiving a functional diagnosis. This process not only prolongs the diagnosis and treatment cycle and increases medical costs but also imposes unnecessary physical and mental burdens on patients (Fikree and Byrne, 2021; Peery et al., 2025). In recent years, with the in - depth understanding of pathological mechanisms such as gastrointestinal motility disorders, visceral hypersensitivity, and gut - brain axis dysregulation, the clinical value of gastrointestinal function assessment has been increasingly emphasized (Dudzińska et al., 2023; Vanuytsel et al., 2023; Russo et al., 2025). Its role has gradually shifted from an auxiliary means in the past to a core component of the modern digestive disease diagnosis system (Keller et al., 2018; Kucharzik et al., 2025).

The clinical promotion of traditional gastrointestinal function assessment techniques has long been restricted by multiple practical obstacles. Although gastric emptying scintigraphy (GES) is regarded as the gold standard, it requires the use of radionuclides and the examination process lasts up to 4 h (Parkman et al., 2025). Gastric balloon manometry can accurately evaluate the gastric receptive relaxation function, but it is significantly invasive due to the need for tube placement, making it difficult to be widely applied in routine diagnosis and treatment (Goelen et al., 2020). High - resolution manometry (HRM) imposes relatively high requirements on patients’ tolerance and equipment platforms (Latorre-Rodríguez and Mittal, 2024). There is a significant trade - off between the diagnostic accuracy and patient acceptance of these methods, which has become a key bottleneck restricting the popularization of function assessment. Fortunately, the development of emerging technologies such as flexible electronics, artificial intelligence, and telemedicine has brought new opportunities to break through this dilemma (Bhaskar et al., 2020; Guo et al., 2025; Wu et al., 2025). Body–surface gastric electrogastrogram mapping (BSGM) has significantly improved the spatial resolution and anti - interference ability, compensating for the deficiencies of traditional electrogastrograms (O’Grady et al., 2023). The stable isotope breath test enables the quantitative assessment of gastric emptying without radiation (Mancuso et al., 2021). After the SmartPill was taken off the market, the new - generation wireless motility capsule (WMC) has taken over the task of monitoring the whole - gut transit time (Kuo et al., 2025). The emergence of wearable sensors has, for the first time, achieved continuous and simultaneous recording of symptom manifestations and physiological signals, opening up a new path for individualized function assessment (Stuart et al., 2022).

**FIGURE 1:**
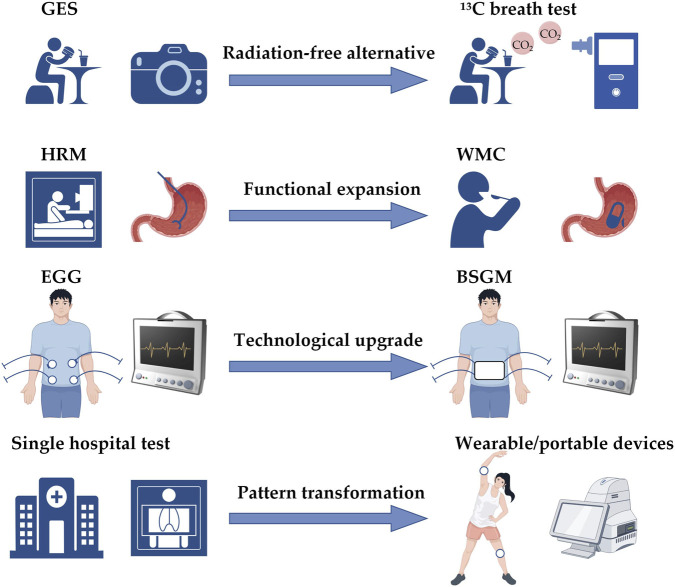
Evolution of gastrointestinal function assessment techniques from traditional models to non-invasive paradigms. GES: Gastric Emptying Scintigraphy; HRM, High-Resolution Manometry; EGG, Electrogastrography; WMC, Wireless Motility Capsule; BSGM, Body Surface Gastric Mapping.

From the core perspective of patient-friendliness, this paper systematically reviews the evolution of gastrointestinal function assessment techniques from traditional methods to non-invasive innovations and their current application status (Figure 1). Specifically, this review aims to answer the following research questions: (1) The limitations of traditional gastrointestinal function assessment techniques in terms of patient - friendliness; (2) When emerging non - invasive technologies improve the patient experience, what are their diagnostic accuracy and clinical value? (3) The potential and limitations of artificial intelligence in the analysis and integration of multi - dimensional physiological signals; (4) The challenges and obstacles faced by the clinical translation of emerging technologies; (5) Whether the direction of technological innovation truly meets the clinical needs of patients. Meanwhile, the main contributions of this review are as follows: (1) Establish a multi - dimensional assessment framework centered on patient experience; (2) Provide a comprehensive comparison and integration across different technology types; (3) Conduct a two - way assessment of technological performance and clinical feasibility; (4) Propose innovation direction guidelines based on patient needs. This paper aims to provide a reference for the rational selection of assessment techniques in clinical practice and offer ideas and inspirations for the future development of this field.

## 2 Limitations in patient-friendliness of traditional assessment techniques

### 2.1 The cost of radionuclide imaging

As the recognized “gold standard” for evaluating gastric emptying function (Maurer and Donahoe, 2024), GES was clearly standardized in the consensus guidelines jointly issued by the American Neurogastroenterology and Motility Society (ANMS) and the Society of Nuclear Medicine (SNM) in 2008. A 4 - hour detection using ^99m^TC - labeled solid meals is adopted, and a gastric retention rate exceeding 10% at 4 h is defined as the standard for delayed gastric emptying (Pal et al., 2023). This method continuously records the radioactive counts in the gastric area through a γ - camera, which can directly and quantitatively reflect the emptying status of gastric contents. Its diagnostic value has been supported by extensive research (Shah et al., 2023).

However, there are concerns about the safety of this technology. During the examination, patients need to ingest food labeled with radioactive substances, with an effective radiation dose of approximately 0.5–2.0 mSv (Knight, 2012). Due to the high sensitivity of fetuses to radiation, pregnant women are absolutely prohibited from undergoing this examination. Children face a higher long - term risk because of their longer life expectancy, and patients with chronic diseases need to consider the cumulative radiation exposure from repeated tests (Lai et al., 2025). From the perspective of patient experience and accessibility, the standard 4 - hour protocol requires patients to stay in the nuclear medicine department for an extended period. Additionally, fasting before the examination and the potentially unpalatable standardized meals may reduce patient compliance (Wise et al., 2021). More importantly, this technology not only relies on dedicated equipment such as expensive γ cameras but also requires professional technicians qualified for the preparation and operation of radiopharmaceuticals (Graham, 2023). Moreover, scintigraphy can only provide a single indicator of gastric emptying rate and cannot cover multi - dimensional information such as gastric regulation, gastric electrical rhythm, or gastric antral contractility. Recent studies have also shown a lack of consistent correlation between delayed gastric emptying and the severity of symptoms, suggesting that relying solely on a single emptying parameter may not be sufficient to fully reflect the complexity of gastric dysfunction (Vijayvargiya et al., 2019).

### 2.2 Trade - off between the depth of information from invasive manometry and the patient burden

The invasive manometry technique reflects the strategy of “prioritizing information depth” in the assessment of gastrointestinal function. Among them, intragastric balloon manometry involves orally inserting a double-lumen catheter with a balloon into the gastric fundus and combining it with a constant-pressure perfusion system to dynamically monitor the pressure-volume relationship. It is currently the reference standard for evaluating gastric regulatory function and can accurately quantify the postprandial relaxation response of the gastric fundus (Gyawali and Kahrilas, 2023). HRM, on the other hand, utilizes a densely arranged array of pressure sensors to record the contractile activities in the gastric antrum, pylorus, and duodenal regions with high spatio-temporal resolution, providing rich physiological information for analyzing the motor coordination of the gastroduodenum (Zheng et al., 2022).

However, this type of technology faces significant challenges in patient tolerance in clinical applications. Gastric balloon manometry requires a catheter to be inserted through the mouth and left in place for several hours. The passage of the catheter through the pharynx often induces obvious nausea and even vomiting reflexes, making it difficult for a considerable number of patients to complete the examination (Leopold et al., 2022; Patel et al., 2024). In children or individuals highly sensitive to stimuli, the operation is often interrupted due to discomfort, and sometimes even mild sedation is required (Oh et al., 2023). Therefore, the invasiveness itself has become the main obstacle to its popularization. From the perspective of accessibility, invasive manometry not only involves expensive equipment and cumbersome operation procedures but also requires high professional capabilities of data interpreters (Kuribayashi et al., 2021). The overall threshold limits its application in routine clinical settings. For this reason, this type of technology is more positioned as a research tool rather than a daily diagnostic method for the general patients, and there is still a large gap before it can become a popular clinical service.

### 2.3 Limitations of traditional non-invasive attempts of electrogastrography

Against the backdrop of radiation exposure in scintigraphy and the invasiveness of manometry, surface electrogastrography (EGG) was once highly anticipated and regarded as a viable approach for non-invasive assessment of gastric function (Popović et al., 2019). This technique records the slow-wave electrical signals of the stomach through electrodes attached to the skin of the upper abdomen, aiming to non-invasively reflect the gastric electrical rhythm and its postprandial changes. However, due to the inherent limitations of the technology, traditional EGG has never gained widespread clinical acceptance (Oczka et al., 2024). It typically uses only a single channel or a few channels for recording, resulting in low spatial resolution. It is difficult to distinguish the electrical activities in different regions of the gastric body, let alone identify abnormalities in the propagation direction of slow waves. In addition, the gastric electrical signals themselves have weak amplitudes and are highly susceptible to interference from physiological and environmental factors such as electrocardiogram, respiratory movement, abdominal wall electromyogram, and body position changes, leading to a low signal-to-noise ratio (Sengottuvel et al., 2018). Coupled with the relatively crude signal processing methods and the high dependence of result interpretation on the operator’s subjective experience, the reproducibility and objectivity of EGG are further undermined.

The above issues lead to inconsistent associations between the indicators obtained by EGG and gastric emptying function as well as clinical symptoms in different studies. Moreover, there is a lack of uniformity in detection protocols, parameter definitions, and interpretation criteria among different centers, showing significant heterogeneity. Therefore, despite its non - invasive advantage, EGG has failed to become a mainstream clinical practice due to its insufficient diagnostic efficacy (Calder et al., 2020).

In summary, traditional gastrointestinal function assessment techniques often have to sacrifice patient comfort, safety, and the accessibility of examinations in order to pursue diagnostic accuracy (Table 1). Moreover, such assessments usually rely on single in - hospital examinations, making it difficult to reflect the dynamic characteristics of symptom fluctuations and physiological changes in functional gastrointestinal diseases. Therefore, their guiding value in real - world clinical scenarios is limited. It is precisely these limitations that have driven the exploration and development of patient - centered, non - invasive novel assessment methods.

**TABLE 1:** Comparison of traditional GI function assessment technology accessibility across economic regions.

Comparison dimensions	HICs	MICs	LICs	Source
NM imaging equipment (units/million population)	1–15	<1	Mostly unavailable	Bour et al. (2022), World Health Organisation (2025)
NM physicians (people/per million population)	∼10	Severely insuficient	Nearly absent	International AtomicEnergy Agency (2019)
HRM availability	Widely available in tertiary centers	Limited to major urban centers	Generally unavailable	(Mariani et al., 2017; International AtomicEnergy Agency, 2019)
Basic imaging access	Near-universal	Limited urban coverage	∼2/3 population lack access	Mariani et al. (2017)
Representative countries	United States of America, Japan Germany France, United Kingdom	China, Brazil India, Mexico	Sub-Saharan Africa. Myanmar	THE WORLD BANK (2025)

HICs, High-Income Countries; MICs, Middle-Income Countries; LICs, Low-Income Countries.

## 3 Breakthroughs in non-invasive technologies

### 3.1 Non - radiation substitution value of stable isotope breath test

The stable isotope breath test (^13^C breath test) is a method to estimate the gastric emptying rate by monitoring the exhaled volume of ^13^CO_2_ produced after the metabolism of ^13^C-labeled substrates. After patients ingest a standard test meal containing ^13^C-labeled substances, the substrates enter the small intestine through the stomach, are absorbed and metabolized, and the generated ^13^CO_2_ is exhaled through respiration. By analyzing these gases using a mass spectrometer or infrared spectroscopy, the kinetic parameters of gastric emptying can be calculated (von Gerichten et al., 2022). According to the different substrates selected, there are currently two main protocols in clinical applications: the ^13^C-octanoic acid breath test and the ^13^C-spirulina breath test. The ^13^C-octanoic acid breath test was first used in clinical practice, and Ghoos et al. established its basic methodological framework (Ghoos et al., 1993). It has been widely used in Europe and the Asia-Pacific region (Keller et al., 2021). In contrast, the ^13^C-spirulina breath test has a more comprehensive regulatory pathway in the United States. It received pre-market approval from the FDA in 2015, was included in Medicare coverage in 2020, and a home sampling version was launched in 2021. Clinical studies (Szarka et al., 2008) have shown that this test has a good correlation with the gastric emptying time measured by scintigraphy (r = 0.73–0.82), with a sensitivity of 83% and a specificity of 81% for diagnosing gastroparesis.

The main advantages of this technology lie in its high safety and simple operation process. As a stable isotope, ^13^C does not produce ionizing radiation, making it suitable for pregnant women, children, and patients with chronic diseases who require repeated testing (Baumann-Durchschein et al., 2022). Sample collection can be carried out in outpatient clinics or even at home, reducing the dependence on medical facilities. However, similar to scintigraphy, the breath test mainly reflects the gastric emptying rate and it is difficult to provide multi - dimensional information. In addition, the test results may be affected by liver metabolism, pulmonary ventilation, and small intestine absorption status (Sanaka et al., 2008; Holzhütter et al., 2020; Mucinski et al., 2023). Therefore, for patients with diseases such as liver cirrhosis, chronic obstructive pulmonary disease, or short bowel syndrome, extra caution is needed when interpreting the results. Differences in substrate types, test meal compositions, sampling times, and calculation models among different commercial platforms also affect the cross - platform consistency and the unity of promotion.

### 3.2 Non-invasive deep detection of body surface and ingestible technologies

#### 3.2.1 Evolution of surface electrogastrography mapping from single-point to global

BSGM has undergone systematic technological upgrades based on EGG, using a high - density electrode array (usually with an 8 × 8 or higher channel) to cover the upper abdomen. By combining advanced spatial filtering and signal processing algorithms, it can effectively separate the slow - wave signals originating from the stomach from artifacts such as electrocardiogram, respiration, and abdominal wall electromyogram, thus achieving multi - dimensional quantification of the frequency, amplitude, propagation speed, direction, and rhythm stability of gastric slow waves (Du et al., 2018; Sukasem et al., 2022; Gharibans et al., 2023). In recent years, BSGM has gradually moved towards clinical translation. The Gastric Alimetry system obtained FDA 510(k) clearance and CE certification in 2022. The international expert consensus led by O’Grady et al. further standardized electrode placement methods, recording durations, standardized test meal protocols, and quality control procedures, laying the foundation for multi - center applications (O’Grady et al., 2023). In terms of clinical research, the Gharibans team found that the incidence of slow - wave rhythm disorders and abnormal propagation in patients with functional dyspepsia and gastroparesis was significantly higher than that in healthy controls (Gharibans et al., 2019). Varghese et al. established the normal reference range of electrogastrographic parameters based on 256 healthy volunteers, providing an important benchmark for clinical interpretation (Varghese et al., 2023a).

BSGM is non-invasive throughout the process, easy to operate, and the equipment is portable, supporting use in outpatient clinics. Different from traditional methods such as gastric emptying scintigraphy, BSGM directly records the electrical activity of gastric slow waves and allows patients to mark symptomatic events, such as nausea or bloating, in real - time during the examination, thus precisely correlating the time points of symptom occurrence with the corresponding gastric electrical patterns at that time. This feature helps to alleviate the common problem in traditional gastric emptying studies, where symptoms are often disconnected from physiological indicators. Traditional methods usually rely on retrospective questionnaires filled out after the examination, making it difficult to accurately restore the specific time and dynamic changes of symptom onset. By continuously monitoring the pre - and post - meal processes in patients’ natural eating states, BSGM not only improves the ecological validity and clinical practicality of the assessment but also constructs a more patient - centered diagnosis and treatment evaluation framework (Humphrey et al., 2025). Meanwhile, modern BSGM systems have significantly evolved from traditional EGG. By integrating automated signal processing and standardized clinical phenotype recognition processes, the interpretation of results has been greatly simplified (O’Grady et al., 2023). However, at present, BSGM still has several limitations. Its monitoring range is limited to the stomach only, and the functions of the small intestine and colon need to be evaluated by other means (O’Grady et al., 2023). The cost of the equipment and disposable electrodes is relatively high, and they have not yet been widely popularized (Erickson et al., 2023; Xu W. et al., 2024). Most of the existing diagnostic thresholds are derived from small-sample cohort studies. Their value in guiding individualized treatment choices and predicting therapeutic effects still awaits verification through large-scale prospective intervention studies (Law et al., 2024).

#### 3.2.2 Full - range monitoring ability of the wireless motility capsule

WMC integrates pH, temperature, and pressure sensors into a swallowable capsule, continuously collecting multimodal physiological data within the digestive tract lumen as it is naturally propelled by gastrointestinal peristalsis (Min et al., 2025). After gastric emptying, the intraluminal pH rapidly changes from acidic to neutral; when the capsule passes through the ileocecal valve into the colon, the pH drops again. These characteristic turning points are used as physiological markers to divide the transit times of the stomach, small intestine, and colon, enabling a segmented assessment of the motor function of the entire digestive tract (Thwaites et al., 2025). The SmartPill has long dominated this field, but this product ceased production in 2023. As a new-generation alternative, the Atmo Gas Capsule received FDA 510(k) clearance in 2025. In addition to inheriting the original sensing functions, it also adds the ability to monitor intraluminal gas components (including hydrogen, carbon dioxide, and oxygen) in real time, providing a new dimension for evaluating intestinal fermentation activity and the metabolic state of microorganisms (Kuo et al., 2025).

The core advantage of WMC lies in the ability to synchronously obtain the transit information of the stomach, small intestine, and colon with just a single oral administration. Moreover, the entire monitoring process does not restrict the patients’ daily activities and involves no exposure to ionizing radiation (Cummins, 2021). However, it is not suitable for patients with dysphagia, known or suspected gastrointestinal stenosis, fistula formation, or those who have recently undergone abdominal surgery. Additionally, in clinical practice, there are occasional cases of swallowing failure, device signal interruption, or capsule retention. In rare cases, endoscopic intervention is required for removal (Gaisinskaya et al., 2022; Schelde-Olesen et al., 2023). However, in individuals with a patent gastrointestinal tract, the incidence of such events is less than 2% (Lee et al., 2014).

### 3.3 Dynamic tracking value of continuous monitoring and remote evaluation programs

Continuous monitoring and remote evaluation utilize wearable or portable devices to extend the collection of physiological data from hospitals to patients’ daily living environments (Anikwe et al., 2022). Currently, several types of technologies have entered the clinical or pre - validation stage. For example, wearable abdominal acoustic sensors (such as the AGIS system) can reflect intestinal peristalsis by recording bowel sounds and have been approved by the FDA for postoperative intestinal function monitoring, with an accuracy rate of up to 97% in predicting intestinal obstruction (Kaneshiro et al., 2016). Heart rate variability (HRV) analysis based on consumer devices such as smartwatches is also used to track autonomic nerve activity, and some studies have shown that it can predict the recurrence of inflammatory bowel disease several weeks in advance (Oakman et al., 2021; Baronetto et al., 2025; Hirten et al., 2025). In addition, some emerging platforms (such as IBD AWARE) attempt to detect inflammatory factors in sweat. Although still in the early exploration stage, they show the potential for home - based dynamic monitoring (Singh et al., 2025).

However, such solutions still face multiple challenges in practical applications. Signals collected from the body surface are easily affected by motion, loose wearing, or environmental interference, and the data quality largely depends on whether patients use the devices properly (Böttcher et al., 2022; Van Der Donckt et al., 2024). Meanwhile, the large amount of continuously generated data lacks unified processing standards and clinical interpretation thresholds, resulting in low interpretation efficiency. More importantly, the current regulatory system has not yet established clear norms for the effectiveness verification, data security, and clinical integration path of such new digital health tools (Ravizza et al., 2019). Therefore, although remote continuous monitoring aligns with the dynamic characteristics of functional diseases, further improvements are needed in terms of technical reliability, practicality, and institutional support before it can be truly implemented in clinical practice.

Currently, non-invasive gastrointestinal function assessment has developed technical paths with different focuses (Table 2), yet they generally share a common development direction. The past practice of relying on single indicators is being replaced by the integration of multi-dimensional physiological signals, and static snapshot-style detections are gradually giving way to long-term dynamic monitoring of gastrointestinal function in real-life environments. This transformation has led to a significant increase in data volume and complexity, far exceeding the capabilities of traditional manual analysis. Efficiently processing and accurately interpreting these high-dimensional and heterogeneous data has become a key bottleneck in promoting the real integration of new technologies into clinical practice, and has also made intelligent analysis tools an urgent need rather than an optional choice.

**TABLE 2:** Comparison of traditional gastrointestinal function and emerging non-invasive gastrointestinal function assessment technologies.

Traditional assessment techniques				Non-invasive technologies			
Dimension	GES	Invasive manometry	EGG	^13^C stable isotope breath test	BSGM	WMC	Wearable sensors
Detection principle	Radioactive nuclide tracking of gastric emptying	Pressure sensors record the intracavity pressure	Recording of gastric slow waves using body surface electrodes	^13^CO_2_ exhalation curve analysis	High-density electrode array recording gastric slow waves	Intraluminal pH/temperature/pressure/gas sensing	Acoustic/HRV/sweat biomarker monitoring
Radiation exposure	Yes	None	None	None	None	None	None
Invasiveness	Low (oral radioactive meal)	High (intubation required)	None (surface electrodes)	None	None	Low (swallow the capsule)	None
Test duration	4 h	Several hours	30 min – several hours	several hours	30 min-several hours	Several days (whole-gut transit)	Days to weeks
Main parameters	gastric emptying rate	pressure-volume, contractile coordination	slow wave frequency	Gastric emptying rate	Slow wave frequency, amplitude, propagation direction, rhythm stability	pH, pressure, temperature, Segmental transit times, intraluminal gas composition	Bowel sounds, HRV, inflammatory markers
Monitoring environment	Nuclear medicine department	Tertiary hospitals or research centers	Outpatient department	Outpatient department	Outpatient department	Outpatient department/In daily life	In daily life (continuous monitoring)
Population restrictions	Contraindicated in pregnancy; caution in children	Poor tolerance patients difficult to complete	No significant restrictions	No significant restrictions	No significant restrictions	Dysphagia/suspected gastrointestinal stricture/gastrointestinal fistula/recent abdominal surgery patients	Individuals with sensitive or allergic skin
Main advantages	Gold standard	Multidimensional functional information	Non-invasive and low-cost	Safe, simple, low cost	Multi-parameter phenotyping, symptom-signal synchronization	Whole-GI coverage, single administration	Dynamic continuous monitoring, captures symptom fluctuations
Main limitations	Radiation, time-consuming, facility-dependent	Poor patient tolerance, high cost	Low spatial resolution, poor signal-to-noise ratio	Affected by hepatic/pulmonary function, platform variability	Limited to stomach only, relatively high cost	capsule retention risk	Signal interference, lack of standards, insufficient validation

## 4 Integration of artificial intelligence and computational methods

### 4.1 Algorithmic innovations in signal analysis and diagnostic pattern recognition

With the development of gastrointestinal function assessment technology towards multi-dimensional, continuous, and high-density data, traditional manual analysis has become increasingly inadequate, driving rapid iteration of signal processing and diagnostic modeling methods (Yang and Bang, 2019; Kröner et al., 2021). In the field of BSGM, researchers have begun to apply convolutional neural networks (CNNs) and recurrent neural networks (RNNs) to automatically recognize the propagation patterns of gastric slow waves (Agrusa et al., 2020). For example, the deep learning algorithm embedded in the Gastric Alimetry system can eliminate artifacts such as electrocardiogram and electromyogram in real time and accurately extract parameters including slow - wave frequency, direction, and rhythm stability. This shortens the manual processing time from several hours to just a few minutes, significantly improving the interpretation efficiency and result consistency (Agrusa et al., 2020; Calder et al., 2022). Practice has shown that customizing deep learning architectures for specific clinical scenarios can significantly enhance the parsing ability of medical artificial intelligence in complex physiological signals (Xiao et al., 2022; Xu T. et al., 2024). Wireless motility capsules can synchronously record multiple signals such as pH, pressure, temperature, and even gas composition. Analyzing single parameters in isolation may lead to overlooking abnormal correlations between different physiological dimensions (Zhou et al., 2024). To address this, researchers have adopted a multi-modal fusion strategy in machine learning, jointly modeling multi-source signals to identify complex functional disorder patterns, such as “normal pressure rhythm but abnormally elevated hydrogen production.” Similarly, long-term data generated by continuous monitoring (such as weeks of heart rate variability, sleep, and activity records) far exceed human analytical capabilities (Chong and Woo, 2021). Temporal models such as long short-term memory networks (LSTMs) have been used to mine early clues of disease activity changes. The Hirten team successfully issued early warnings several weeks before the onset of clinical symptoms in patients with inflammatory bowel disease using such methods (Hirten et al., 2025).

Signal processing still faces significant technical challenges. Due to the weak nature of EGG signals, they are highly susceptible to interference from electrocardiogram and electromyogram artifacts. In conventional analysis, 36.5% of the recording periods are often excluded, severely limiting the data availability (Schamberg et al., 2023). BSGM effectively improves the signal quality through a high - density electrode array, individualized electrode placement, and an automated processing workflow. Meanwhile, the automatic artifact detection algorithm developed by Calder et al. achieved both sensitivity and specificity exceeding 95% when compared with expert annotations. The Fleiss’ Kappa consistency among experts was 0.82, indicating a high level of reliability in the algorithm’s interpretation (Calder et al., 2022). Preliminary studies have demonstrated the potential of algorithms in enhancing diagnostic performance. However, it should be noted that most current studies are still based on retrospective or single - center data. The generalization ability of the algorithms across different device platforms and diverse populations remains unclear and urgently needs to be verified through multi - center, prospective studies.

### 4.2 Construction of interpretability and clinical decision support

No matter how advanced an algorithm is, it is difficult to apply clinically if doctors cannot understand its judgment basis (London, 2019). In the past, many AI models were like “black boxes”, providing only conclusions without reasons. Doctors could neither confirm the reliability of the results nor trace errors when they occurred. This opacity makes it difficult for clinical practice and supervision to accept (Yang et al., 2022; Muhammad and Bendechache, 2024). Currently, AI in the field of gastrointestinal function assessment is shifting towards interpretability (Yuan et al., 2024): some systems can show which parameters have the greatest impact on the diagnosis, and some use visualization techniques to mark the signal segments that the model focuses on (Auzine et al., 2024), relating the conclusions to specific physiological changes such as “decreased slow-wave frequency” or “abnormal propagation direction”. Expert consensus and regulatory guidelines also clearly state that algorithm outputs must be presented in a clinically understandable manner, and manufacturers need to explain the decision-making logic and continuously monitor the performance (Holm, 2022; Clark et al., 2023).

However, interpretability is merely the first step towards clinical implementation. A more profound challenge lies in how artificial intelligence systems can adapt to the highly heterogeneous medical environments in the real world. In tertiary hospitals, high-performance computing resources and professional technical support are relatively abundant. In contrast, in primary healthcare institutions, due to limited computing power, poor network conditions, and a shortage of professional technicians, technologies that rely on cloud processing or frequent updates are often difficult to implement (Lemes et al., 2025). Meanwhile, when the information prompted by the intelligent platform conflicts with the diagnosis of clinical doctors, there is currently a lack of standardized procedures to reconcile such differences in human-machine judgments. Most systems only provide auxiliary information maps, and the final diagnosis is still comprehensively determined by doctors. However, an effective feedback loop has not been established to continuously optimize the accuracy of the model (Gong et al., 2025). Additionally, the long-term operation of these systems also involves issues such as maintenance costs, data security, and technological updates. Once the technical support ceases or hospitals are unable to afford the necessary upgrade fees, these systems may quickly become obsolete. These issues have led to the fact that the applications of many current advanced technologies still remain at the theoretical verification stage and have not truly been integrated into daily clinical practice to improve patient treatment outcomes.

Future research needs to move beyond the mere pursuit of algorithm accuracy and shift towards a design concept that emphasizes practical clinical value. This entails conducting large-scale prospective studies to validate the broad applicability of the technology, developing simplified tools suitable for use in resource-constrained settings, establishing protocols that can effectively integrate the judgments of doctors and computers, and exploring ways to ensure the sustainability of technology updates and services through public or private partnerships (Zhang et al., 2025). Only in this way can these advanced technologies transition from the laboratory to clinical practice, becoming both practical and reliable medical tools, achieving comprehensive progress from accurate diagnosis to effective treatment, and thereby substantially improving the diagnosis and treatment of gastrointestinal dysfunction.

## 5 From technical validation to clinical decision-making: transformation pathways and implementation challenges

### 5.1 From diagnostic accuracy to clinical utility

Traditionally, the accuracy of new technologies is often evaluated by comparison with the so - called “gold standard”, with the assumption that the more consistent the results, the more reliable the technology. However, in the field of gastrointestinal function assessment, the “gold standard” itself has limitations. Studies indicated that the correlation between delayed gastric emptying measured by scintigraphy and the severity of patients’ symptoms was not strong. This implies that patients with abnormal test results may not have obvious symptoms, while those with severe symptoms may have normal test results (Vijayvargiya et al., 2019; Camilleri et al., 2023). If a “gold standard” is divorced from clinical reality, even if a new technology can perfectly replicate these results, its contribution to clinical decision - making is limited (Table 3). These new technologies are gradually demonstrating their value in clinical management. Recent studies have shown that electrophysiological classification based on BSGM may contribute to optimizing treatment options in specific patients. Different from the traditional approach that relies on symptoms and tries drugs step - by - step, BSGM can reveal specific abnormal patterns of gastric slow - wave activity and correlate them with the clinical situation. Taking the treatment of gastroparesis as an example, when BSGM detects unstable gastric antral slow - wave frequency (such as frequent tachycardia or bradycardia), it indicates a possible disorder of neuroelectrical activity. In this case, increasing prokinetic drugs often yields poor results, and neuromodulation therapies such as gastric electrical pacing should be prioritized. If the slow - wave frequency is stable but the amplitude is reduced and the conduction is abnormal, a dietary adjustment strategy may be more appropriate (Camilleri, 2016; Gharibans et al., 2022; Varghese et al., 2025). In the management of chronic constipation, the traditional method mainly selects medications empirically based on symptom characteristics, often requiring multiple medication changes. WMC can measure the transit time of each segment at once, distinguishing between types dominated by delayed gastric emptying or slow colonic transit, thereby guiding the choice of prokinetic drugs or secretagogues. This stratified treatment strategy based on the transit pattern has been incorporated into the ACG gastroparesis guidelines.

**TABLE 3:** Diagnostic accuracy analysis of gastrointestinal motility assessment techniques.

Technologies	Sensitivity (%)	Specificity (%)	CV(%)^*^	Source
GES	92	80	8–31	(Abell et al., 2008; Hou et al., 2011)
Invasive manometry	Achalasia: 93–98	96–98	15	(Bogte et al., 2011; van Hoeij et al., 2015; Latorre-Rodríguez and Mittal, 2024)
	Gastroesophageal reflux: 65–70	80–85		
EGG	55–60	65–80	3.7–30	(Chen and Lin, 1994; Krusiec-Swidergoł and Jonderko, 2008; Riezzo et al., 2013)
^13^C Stable isotope breath test	67–75	80–86	16.3–29.6	(Ziegler et al., 1996; Choi et al., 1997; Kasicka-Jonderko et al., 2006)
BSGM^**^	Not established	Not established	0.2–1.9	Law et al. (2024)
WMC	59–86	64–81	13–28	(Stein et al., 2013; Thwaites et al., 2022)
Wearable sensors	80–88	83–88	Not established	Baronetto et al. (2025)

*CV, Coefficient of Variation- The ratio of the standard deviation to the mean, used to measure the relative dispersion of data. A lower value indicates less relative fluctuation of the data and more concentrated and stable data.

**BSGM, The sensitivity and specificity cannot be established due to the lack of a direct comparison. In addition, the limitations of the traditional gold standard also cast doubt on the applicability of these indicators in this field. Nevertheless, compared with traditional assessment methods, BSGM, has shown a higher diagnostic rate (Wang et al., 2019).

This transformation reflects an important adjustment in the thinking of technological verification. According to the diagnostic technology evaluation model proposed by Fryback and Thornbury (Fryback and Thornbury, 1991; Thornbury, 1994), technological verification can be divided into five levels: the lowest level focuses on the technical efficacy and diagnostic accuracy of the device, namely, the stability of the device and its ability to distinguish diseases; above it are diagnostic thinking efficacy, treatment efficacy, and patient outcome efficacy in sequence, which respectively examine whether the test results can influence doctors’ judgments, the selection of treatment plans, and ultimately whether the patients’ health conditions can be improved. Currently, most research on new technologies mainly focuses on the first two levels, but it is the latter three levels that truly reflect the clinical value. The formation of this phenomenon stems from multiple deep-seated reasons. Firstly, ethical concerns about the potential risks that unproven new technologies may pose to patients often limit research designs to the validation of diagnostic accuracy. Secondly, long-term prognosis studies usually require several years of follow-up, while the funding cycles of most scientific research projects are only three to 5 years. Moreover, the current academic evaluation system favors short-term achievements. Coupled with the obvious symptom fluctuations and complex natural courses of functional gastrointestinal diseases, the feasibility of establishing clear causal relationships is further weakened. Thirdly, in the early stage of technology transformation, researchers tend to choose diagnostic accuracy studies that use existing gold standards as references and have clear and controllable endpoints, while deliberately avoiding efficacy or outcome studies involving complex clinical situations, multiple confounding factors, and individualized decision-making processes (Stackland et al., 2025). These factors are interwoven, resulting in the current evidence-based evidence mostly focusing on the front-end process of technical performance verification and being insufficient in supporting its real-world clinical utility. It must be acknowledged that there are still obvious gaps in the evidence chain from diagnostic accuracy to actual management outcomes. Such data gaps are inevitable in the process of any new technology moving towards clinical application, as the accumulation of real-world evidence takes time. It usually takes five to 10 years to clarify through prospective studies whether new indicators can truly change diagnosis and treatment decisions and ultimately improve patient prognosis. Currently, most studies still focus on validating the diagnostic performance of the technology itself, and there are limited randomized controlled trials that truly compare the advantages and disadvantages of technology-guided treatment with traditional regimens. This is not to deny the value of these new technologies, but to remind us to maintain scientific prudence. Their exact clinical status remains to be gradually established through more utility studies and real-world data, and more adaptable research designs need to be explored in the future. Pragmatic randomized controlled trials can compare changes in treatment decisions before and after technology application in real clinical settings, improving the external applicability of results while ensuring internal validity. Real-world studies based on electronic medical records and disease registration systems can accumulate large-scale, long-term follow-up data at a relatively low cost. In addition, establishing a multi-center registration system to systematically collect application data of various technologies in different clinical settings, including diagnostic results, treatment decisions, follow-up outcomes, and adverse events, will provide a solid foundation for subsequent evidence-based evaluations.

The verification of new technologies should not merely focus on the accuracy of measurement results, but should more comprehensively consider whether this measurement can assist doctors in making better clinical decisions and ultimately whether patients can benefit from it (Bakker et al., 2025). Given the special challenges in the field of gastrointestinal function assessment, such as the frequent mismatch between symptoms and test results and the large fluctuations in functional status, it is necessary to adopt a multi - dimensional evaluation method, pay attention to the individual longitudinal changes of patients, and incorporate patient - reported outcome data, so as to establish a solid evidence base for clinical utility beyond traditional diagnostic accuracy (Tack et al., 2024).

### 5.2 Integration of clinical decision pathways

Currently, some medical institutions have begun to use the ^13^C breath test to replace scintigraphy for gastroparesis screening. This shift has significantly improved the accessibility and safety of the examination, especially for pregnant women, children, and primary - level units lacking nuclear medicine facilities (Keller et al., 2018; Karrento et al., 2025). The Spiegel team applied AGIS abdominal acoustic monitoring for real - time assessment of postoperative intestinal function recovery, which helps the surgical team promptly identify patients at high risk of delayed intestinal function recovery (Kaneshiro et al., 2016). Changes in heart rate variability (HRV) in patients with inflammatory bowel disease (IBD) often occur earlier than clinical symptoms, suggesting that continuous physiological monitoring has the potential for early warning (Hirten et al., 2021; 2025; Yerushalmy-Feler et al., 2022).

These practices have revealed two main paths for integrating new functional assessment technologies into clinical practice. The first is triage - through non - invasive initial screening, it helps doctors determine which patients need further invasive examinations and which can directly start empirical treatment, thereby optimizing the diagnosis and treatment process and resource allocation. The second is monitoring - using repeatable and non - invasive methods to dynamically track treatment responses or predict disease activity. However, there is currently insufficient evidence to suggest that functional assessment results can directly guide specific treatment choices. For example, the early warning potential of HRV monitoring in inflammatory bowel disease is highly anticipated. However, whether it can truly optimize the treatment timing and reduce the risk of recurrence needs to be verified through prospective randomized controlled trials.

### 5.3 Realistic obstacles to implementation and transformation

Even if a technology has passed clinical verification, it does not necessarily mean that it can be smoothly integrated into daily diagnosis and treatment. Take the ^13^C-spirulina breath test as an example. It was approved by the FDA as early as 2015, but was not included in the Medicare reimbursement scope until 2020. During these 5 years, despite the availability of the technology, it was difficult to promote because patients had to pay out of pocket (Sexton et al., 2023). Similarly, although the Gastric Alimetry system obtained FDA 510(k) clearance in 2023, it is currently only listed as an “optional” option rather than a clear recommendation in the mainstream gastrointestinal motility guidelines (Camilleri et al., 2022). The BSGM international consensus led by O’Grady et al. provides a standardized framework for electrode placement, quality control, and result interpretation. However, it also reflects the obvious shortcomings in operational standardization and professional capacity building in this field, which urgently need to be filled through consensus guidance (O’Grady et al., 2023). Simultaneously, establishing a comprehensive and standardized reference range remains a significant challenge for achieving widespread clinical application. Although recent studies have made some progress (Wang et al., 2015; Karrento et al., 2025), such as a 2015 study on WMC transit time that identified the influence of gender and age, and the pediatric normal values for the ^13^C-breath test established in 2025 based on 301 children, which confirmed the impact of gender and pubertal development status, these data are still insufficient to support robust population-specific criteria. Currently, most normal value studies focus on Western populations, and there is an obvious lack of evidence regarding racial differences in gastrointestinal transit physiology. Moreover, the influence of dietary patterns in different cultural backgrounds on the reference range has not yet been systematically evaluated. These gaps pose important obstacles to the global promotion of the technology, highlighting the urgent need for international cooperation.

These cases indicate that the successful technical verification is only the first step, and the actual implementation still requires overcoming multiple interrelated real - world obstacles. There is a close relationship among medical insurance coverage, operation standardization, and professional talent cultivation. If no consensus is reached on operation standards and quality control processes, it will be difficult for the medical insurance department to formulate clear reimbursement standards and the scope of indications, making it hard to include the technology in medical insurance coverage. The lack of medical insurance support means that hospitals have insufficient motivation to implement the technology, and patients are reluctant to bear the additional costs, which may lead to the shelving of advanced technologies (Groene and Schneck, 2023). Meanwhile, the shortage of professional talents restricts the promotion speed of the technology. Training qualified personnel requires time and resources, and the limited application scale weakens the enthusiasm for talent cultivation, thus creating a vicious circle. The introduction of new technologies poses new requirements on hospitals from equipment configuration to data management (Borges do Nascimento et al., 2023). Non - standard operations or misinterpretations not only waste resources but may also mislead diagnosis and treatment decisions, affecting the medical insurance department’s confidence in the reliability of the technology. Certainly, to comprehensively evaluate the implementation barriers of these technologies, their health economic values must also be considered. Although the high upfront investment poses a certain threshold, more accurate diagnosis can often reduce repeated examinations and ineffective interventions, resulting in significant savings. When a definitive examination can replace multiple vague or uncertain operations, even if the initial cost is relatively high, the overall medical expenditure may actually decrease. This approach is not only more cost - effective but also more in line with the patient - centered medical concept.

For different technologies, the solutions to transformation barriers need to be customized according to local conditions. For example, the ^13^C breath test is easy to operate and has a low equipment threshold. It is suitable for promoting the construction of a primary - level training system and the formulation of standardized operating procedures, providing a basis for inclusion in the medical insurance. As a high - density electrode array technology, BSGM involves complex data analysis and requires high - level operators. It is necessary to rely on regional training centers and expert certification systems to establish full - process quality control standards. Therefore, it is far from enough for technology developers to work alone. Clinical experts, medical institutions, payers, and regulatory authorities need to form a joint force, jointly promote in aspects such as payment policies, training systems, and usage standards, and formulate differentiated promotion strategies according to the characteristics of the technology. Only in this way can innovation truly move from the laboratory to the consulting room (Qiu et al., 2021; Gutersohn et al., 2023).

## 6 Discussion

This study systematically reviews the technological evolution in the field of gastrointestinal function assessment from the perspective of patient - friendliness, covering the limitations of traditional methods, breakthroughs in new non - invasive technologies, the integrated application of artificial intelligence, and the practical obstacles in clinical promotion. For a long time, there has been an inertial thinking in clinical practice that the more invasive the examination, the more reliable the information. For example, intragastric manometry requiring tube placement is regarded as the gold standard, while non - invasive methods are often downgraded to preliminary screening or sub - optimal choices (Jandee et al., 2022). However, regardless of the safety, invasiveness, and accessibility issues of traditional gastrointestinal function assessment techniques, these techniques generally rely on single - time static in - hospital examinations. The symptoms of functional gastrointestinal diseases are significantly fluctuating and intermittent, and patients may not show typical symptoms on the day of consultation, resulting in the failure to effectively capture physiological abnormalities (Desai et al., 2018; Black et al., 2020). This instantaneous snapshot - style assessment method is difficult to reflect the real dynamic characteristics of the disease, thus weakening the diagnostic sensitivity and clinical relevance. Recent technological advancements are challenging this concept. BSGM can reveal the spatial distribution characteristics of gastric electrical activity without any contact with the inside of the body (Varghese et al., 2023b); WMC, only as small as a pill, can record the movement from the stomach to the colon and the intraluminal environment throughout the process after being swallowed (Gharibans et al., 2022; Zhou et al., 2024); wearable devices can continuously collect physiological signals for days or even weeks, presenting dynamic changes that cannot be captured by a single examination (Chong and Woo, 2021). These innovations indicate that patient comfort and the depth of diagnostic information are not mutually exclusive. Non - invasive technologies are breaking the old either - or framework, providing more abundant and clinically relevant information closer to the real disease course while reducing the burden on patients (Gharibans et al., 2022). Existing clinical evidence preliminarily supports the patient-friendliness of non-invasive techniques. Studies have found that patients undergoing capsule endoscopy generally have better satisfaction and comfort than those undergoing traditional endoscopy. For example, capsule colonoscopy is significantly superior to traditional colonoscopy in terms of pain, with only 2% of patients reporting pain compared to 21% for traditional colonoscopy (Dale et al., 2025). In addition, a real-world study in Scotland showed that more than 80% of patients who underwent capsule colonoscopy were willing to recommend the service to others (Bond et al., 2023). Given the similarity between WMC and diagnostic capsules in terms of administration method and examination experience, these findings suggest that capsule technology may generally have better patient acceptance. At the same time, for other novel gastrointestinal motility assessment techniques, such as ^13^C breath test and BSGM, there is currently a lack of direct comparison data with traditional examination methods in terms of patient satisfaction. Considering the differences between these new technologies and traditional methods in terms of operation procedures, radiation involvement, and overall experience, it is necessary to systematically understand patients’ real feelings towards them in the future, which will be a gap worth filling in related research.

Certainly, this does not imply that non-invasive techniques can completely replace traditional methods. Current non-invasive approaches still have obvious information blind spots, and different technical routes have different focuses (Wang et al., 2023). The stable isotope breath test is mainly used to evaluate gastric emptying function, BSGM focuses on gastric electrical rhythm and its propagation characteristics (Schamberg et al., 2023), WMC can track the transit process of the stomach, small intestine, and colon, and continuous monitoring technology is good at capturing dynamic physiological changes during symptom fluctuations (Gharibans et al., 2022). A single method is difficult to provide a comprehensive assessment of gastrointestinal function, and in clinical applications, it is often necessary to flexibly select or combine methods according to specific goals. Therefore, when dealing with specific patients, doctors still need to make individualized trade-offs based on the characteristics of the condition. Specifically, for pregnant women, children, or patients with chronic diseases requiring repeated monitoring, the ^13^C breath test can assess gastric emptying without radiation exposure. Once its diagnostic accuracy meets clinical requirements, it can completely replace scintigraphy as the first-line examination method. In primary healthcare institutions lacking nuclear medicine facilities, the breath test or BSGM can also be used as a screening tool, effectively alleviating the problem of insufficient service accessibility. However, when the results of non-invasive examinations are inconsistent with clinical manifestations, anatomical abnormalities are suspected, or precise lesion location is needed to guide surgery, traditional invasive techniques are still required for supplementation or confirmation. In clinical practice, a stepwise approach from non-invasive to invasive methods should be adopted. That is, non-invasive methods are used as the initial screening, and then, based on the reliability of the results and specific clinical needs, a transition to more in-depth traditional examinations is made in an orderly manner. This approach can reduce unnecessary examinations and resource consumption while ensuring comprehensive and accurate diagnosis (Fikree and Byrne, 2021; Kucharzik et al., 2025). However, it should be noted that current evaluations of these non-invasive techniques mainly focus on short-term safety (e.g., no ionizing radiation and minimal invasiveness), and long-term follow-up data are lacking. For example, it is still unknown whether the repeated long-term use of substrates labeled with stable isotopes has potential impacts on human metabolism. Similarly, chronic skin irritation or allergic reactions caused by long-term wearing of wearable sensors also deserve attention, especially for patients who need continuous monitoring over a long period (Ravizza et al., 2019). The lack of evidence on long-term safety indicates that the current evaluation of the safety of these techniques is not comprehensive. Future studies should include longer follow-up periods to obtain more complete safety data, which can provide a basis for clinical decision-making.

The ultimate goal of technological innovation is to improve patients’ lives and solve practical problems. However, it is worth reflecting on whether the current progress in the field of gastrointestinal function assessment truly meets the core needs of patients. To date, such innovations have been mostly led by engineers and researchers, focusing on enhancing resolution, expanding the types of parameters, or optimizing algorithm accuracy. Nevertheless, these technological breakthroughs do not necessarily translate into perceptible benefits for patients (Tack et al., 2024). The most pressing demands of patients with functional gastrointestinal disorders are often not to obtain a “precise” diagnostic label, but to find treatment plans that can effectively relieve discomfort and improve their quality of life (Goebel-Stengel et al., 2023). When examining the real needs of patients, we find that their dimensions go far beyond “safety, comfort, and economy”. Patients also care about the comprehensibility of the interpretation of diagnostic results. If complex technical parameters cannot be translated into easy - to - understand language, it is difficult to promote patients’ understanding of their own conditions and treatment compliance. In addition, privacy protection during the technical operation process cannot be ignored, especially for wearable devices and remote monitoring involving continuous data collection. Patients have reasonable concerns about the security of their personal health information. There are significant differences in technology acceptance among different age groups. Elderly patients may be unfamiliar or afraid of operating smart devices, and for child patients, their cooperation and parents’ participation methods need to be considered (Wang et al., 2022). These needs are often marginalized in the design of existing technologies.

From the perspective of human factors engineering, patient - centered technology development should incorporate human cognitive characteristics, behavioral patterns, and usage scenarios into the entire design process (Polhemus et al., 2020). This implies that the technology evaluation framework needs to transcend the traditional single - dimension of “diagnostic accuracy” and establish an effective connection mechanism between technical parameters and patient - reported outcomes. For example, when evaluating BSGM or wireless power capsules, attention should not only be paid to their detection ability for gastric electrical rhythm disorders or abnormal transit times, but also subjective outcome indicators such as the degree of symptom improvement, recovery of daily function, and improvement of quality of life of patients should be systematically included. At the same time, the “usability” of the technology, including interface friendliness, operation learning curve, result presentation mode, etc., also needs to be evaluated to ensure that the technology can be smoothly used by patients with different backgrounds in the real world. Unfortunately, these real - world considerations are often overlooked in the early stage of technology (Drossman et al., 2021). Future research should listen more to the voices of patients, incorporate their experiences, preferences, and concerns into the technology design and evaluation framework, seek a dynamic balance between technical parameter optimization and patient experience improvement, and enable innovation to truly return from parameters to people and from the laboratory to life. In this way, technological innovation can better meet the actual needs of patients and improve their quality of life.

In the future, non-invasive gastrointestinal function assessment is expected to become an important pillar of precision medicine. Artificial intelligence is driving the integration of multimodal data, fusing endoscopic images, histopathology, molecular markers, and patient-reported outcomes to achieve fine classification of functional diseases such as inflammatory bowel disease and prediction of treatment responses (Iacucci et al., 2025). Cutaneous electrogastrogram imaging or continuous monitoring data can also be combined with symptom patterns, psychological states, and lifestyle factors to construct a multi-dimensional phenotype of functional gastrointestinal disorders, breaking through the limitations of traditional one-size-fits-all treatments (Dowrick et al., 2024; Zhu et al., 2024). Meanwhile, the bidirectional interaction between gastrointestinal motility and the gut microbiota provides a new dimension for diagnosis and treatment. Fecal metagenomic analysis and swallowable sampling capsules enable the assessment of regional microbiota, and cutting-edge technologies such as CRISPR-based sentinel bacteria are more expected to achieve real-time monitoring of the dynamic interaction between the host and microorganisms (Waimin et al., 2020; Schmidt et al., 2022). Although most methods are still in the early research stage, and their clinical feasibility, safety, and regulatory pathways still need systematic verification, such cross-scale and multi-system integration strategies are promoting the transformation of gastrointestinal function assessment from descriptive diagnosis to mechanism-driven individualized intervention.

## 7 Conclusion

Gastrointestinal function assessment is at a critical stage of technological transformation. The rapid development of non-invasive methods provides a practical alternative to the limitations of traditional examinations in terms of radiation exposure, operational invasiveness, and service accessibility. The introduction of artificial intelligence has also significantly improved the processing efficiency of physiological signals and the ability of clinical decision support. However, the availability of technology does not necessarily mean its clinical applicability. Currently, most studies still focus on validating diagnostic accuracy, and there is still a lack of high-quality evidence on whether these new technologies can optimize treatment strategies and improve patients’ long-term outcomes. Meanwhile, long-term follow-up data on the potential risks of non-invasive innovative technologies are also lacking, and the safety assessment is incomplete. In the future, it is urgent to establish multi-center, standardized validation datasets and take patients’ actual benefits as the core dimension for technology evaluation.

In addition, the AI-driven multimodal data integration and in-depth exploration of the gut microbiota-motility axis are opening up a new path for gastrointestinal function assessment, shifting from descriptive diagnosis to mechanism-driven individualized intervention. Although most cutting-edge technologies are still in the early research stage and their clinical translation requires systematic validation, the research paradigm of cross-scale and multi-system integration is beginning to emerge. To achieve this vision, it is essential to synchronously build a supporting system during the alternation of old and new technologies, including the adaptive adjustment of clinical diagnosis and treatment processes, standardized training of medical staff, and follow-up of patient education strategies. Only by promoting the synergy of technology, process, and personnel can a smooth transition be achieved. In clinical practice, doctors should weigh the advantages and disadvantages of different technologies based on specific problems, select the most appropriate assessment path, and maintain necessary prudence while actively embracing innovation.
